# α-Synuclein Negatively Regulates Nurr1 Expression Through NF-κB-Related Mechanism

**DOI:** 10.3389/fnmol.2020.00064

**Published:** 2020-05-12

**Authors:** Congcong Jia, Hongqian Qi, Cheng Cheng, Xuefei Wu, Zhaofei Yang, Huaibin Cai, Sheng Chen, Weidong Le

**Affiliations:** ^1^Center for Clinical Research on Neurological Diseases, The First Affiliated Hospital, Dalian Medical University, Dalian, China; ^2^Liaoning Provincial Key Laboratory for Research on the Pathogenic Mechanisms of Neurological Diseases, The First Affiliated Hospital, Dalian Medical University, Dalian, China; ^3^Liaoning Provincial Key Laboratory of Cerebral Diseases, Department of Physiology, College of Basic Medical Sciences, Dalian Medical University, Dalian, China; ^4^Transgenic Section, Laboratory of Neurogenetics, National Institute on Aging (NIA), National Institutes of Health, Bethesda, MD, United States; ^5^Department of Neurology, Ruijin Hospital, Shanghai Jiao Tong University School of Medicine, Shanghai, China

**Keywords:** α-synuclein, nuclear factor κ B (NF-κB), nuclear receptor-related 1 protein (Nurr1), Parkinson’s disease, dopamine

## Abstract

The nuclear receptor-related 1 protein (Nurr1) is critical for the development and survival of midbrain dopamine neurons that are predominantly affected and progressively degenerated in Parkinson’s disease (PD). The expression level of Nurr1 has been proposed to be modulated by α-synuclein (α-SYN), an important pathological hallmark of PD. However, the underlying molecular mechanisms of α-SYN-Nurr1 interaction are still rarely explored. In this study, we investigated the effect and mechanism of α-SYN on the transcription level of Nurr1. Our results showed that overexpression of α-SYN (WT or A53T) reduced Nurr1 and its downstream gene expressions. α-SYN neither affected the mRNA stability nor bound with the promoter of Nurr1, but modulated the transcription activity of Nurr1 promoter region ranging from −605 bp to −418 bp, which contains the binding site of nuclear factor-kappa B (NF-κB). Moreover, overexpression of α-SYN (WT or A53T) down-regulated NF-κB expression level, thereby inhibiting the transcription factor activity of NF-κB and decreasing the binding quantity of NF-κB with Nurr1 promoter. These findings may give us new insights to better understand the molecular mechanisms underlying the α-SYN-regulated Nurr1 function, which may fascinate the investigation of dopamine neuron degeneration in PD pathogenesis.

## Introduction

Parkinson’s disease (PD) is pathologically characterized by the progressive loss of dopamine (DA) neurons in the substantia nigra and the presence of intracellular inclusions in the remaining nigral neurons, named Lewy bodies (LBs; Lees et al., 2009). α-Synuclein (α-SYN) and its aggregates have been found as the main components of LBs (Spillantini et al., 1997; Dong et al., 2016). Human α-SYN is a 140-amino acid protein, which is mainly expressed in neurons. α-SYN forms toxic oligomers, protofibrils or fibrils, affects DA neuron function, and causes neurodegeneration (Dev et al., 2003). Moreover, various mutations, such as A53T, A30P, and E46K, have been reported to influence the formation of protofibril or fibril of α-SYN (Krüger et al., 1998), and they are associated with autosomal dominant familial type of PD (Polymeropoulos et al., 1997).

The nuclear receptor-related-1 protein (Nurr1, also known as NR4A2), a member of the inducible orphan nuclear receptor family (Wang et al., 2003), is critical for the development and survival of midbrain DA neurons, which are predominantly affected and progressively degenerate in PD (Saucedo-Cardenas et al., 1998; Le et al., 1999; Jiang et al., 2005; Smidt and Burbach, 2007). Previous studies from our group and others have indicated that the absence of Nurr1 causes defects in DA neuron development and functional maintenance (Le et al., 1999; Jiang et al., 2005; Kadkhodaei et al., 2009, 2013). We also found that the mutations in the first exon of NURR1 gene (−291Tdel and −245T → G) are associated with human familial PD (Le et al., 2003). Therefore, Nurr1 has been regarded as a potential susceptibility gene for PD (Bruning et al., 2019). This gene may also serve as a master downstream molecular target for α-SYN-induced neuron toxicity (Decressac et al., 2012). Excess cellular concentrations of α-SYN effectively block the trophic response of DA neurons to glial cell-derived neurotrophic factor (GDNF) *via* reducing expression of Nurr1 (Decressac et al., 2012). Lin et al. (2012) have found that conditional expression of mutant α-SYN in the midbrain DA neurons caused Nurr1 degradation and progressive neurodegeneration. However, the effects and the exact molecular mechanisms of α-SYN affecting Nurr1 transcription level are rarely investigated.

A previous study has suggested that the Nurr1 promoter region contains the binding site of nuclear factor-kappa B (NF-κB), indicating the regulation of NF-κB on Nurr1 expression (Ichinose et al., 1999). Prostaglandin E2 (PGE2) stimulation of the human EP1 receptor up-regulates the expression of Nurr1 by activation of the NF-κB signaling pathway (Ji et al., 2012). Moreover, inflammatory mediators can enhance NF-κB-binding activity with the Nurr1 promoter, which in turn promote Nurr1 transcription and markedly elevate Nurr1 mRNA and protein levels (McEvoy et al., 2002). All these findings may suggest the potential involvement of NF-κB in α-SYN-regulated Nurr1 expression.

In this study, we evaluated the effects of α-SYN on expression levels of Nurr1 and its downstream genes. We also explored the mechanism of α-SYN on Nurr1 transcription level, by investigating the effects of α-SYN on Nurr1 mRNA stability, and screening the potential Nurr1 promoter region affected by α-SYN. Our results showed that α-SYN (both WT and A53T) did not affect Nurr1 mRNA stability, but modulated the transcription activity of Nurr1 promoter fragment (ranging from −605 bp to −418 bp). Then, we identified NF-κB, the highest score transcription factor of Nurr1, by analyzing α-SYN-regulated Nurr1 promoter region with JASPER database, and detected the binding quantity of NF-κB with this region in α-SYN overexpressed cells showing that α-SYN regulated Nurr1 expression *via* affecting the binding quantity of NF-κB with Nurr1 promoter region. Taken together, these results demonstrate a NF-κB-related mechanism underlying the regulating effect of α-SYN on Nurr1 expression.

## Materials and Methods

### Cell Culture and Transfection

Mouse neuroblastoma (N2a) cells were cultured in Dulbecco’s modified Eagle’s medium (DMEM; Gibco, MA, USA) containing 10% fetal bovine serum (Gibco) and 1% penicillin/streptomycin solution (Sigma–Adrich, St. Louis, MO, USA) in humidified atmosphere incubator at 37°C with 95% air and 5% CO_2_.

Human α-SYN^WT^ [pcDNA3.1(-)-SYNC], α-SYN^A53T^ [pcDNA3.1(-)-A53T] or empty vector [pcDNA3.1(-)] plasmids (2 μg) were transfected into N2a cells with lipofectamine 6,000 reagents (Beyotime, Shanghai, China) according to the manufacturer’s instructions, respectively. After transfection for 72 h, cells were cultured with medium containing Geneticin (600 μg/ml; Thermo Fisher Scientific, Waltham, MA, USA) to obtain α-SYN stably overexpressed cells. Western blot was used to detect the expression level of α-SYN in these cells. Human Nurr1 [pcDNA3.1(-)-Nurr1] or pcDNA-3.1 plasmids (2 μg) were transfected into α-SYN^WT^ or α-SYN^A53T^ overexpressed cells for 72 h with lipofectamine 6,000 reagents (Beyotime) according to the manufacturer’s instructions, respectively.

### Western Blot

For analysis of protein expression, cells were washed with phosphate-buffered saline (PBS), re-suspended in RIPA buffer (Beyotime), incubated on ice for 30 min, and then centrifuged at 12,000 rpm for 15 min. The supernatant was used to detect the concentration of protein with a BCA Protein Assay kit (Beyotime). The remaining supernatant was added with 5× sodium dodecyl sulfate (SDS, Beyotime) sample buffer and boiled for 10 min. Then, equal amounts of total protein lysates (30–50 μg) were separated by 8–15% SDS-polyacrylamide gel electrophoresis (PAGE) and transferred onto 0.45 μm polyvinylidene difluoride (PVDF) membranes (Millipore, Kankakee, MA, USA). After blocking with 5% skimmed milk for 1 h at room temperature, the membranes were incubated with the primary antibody at 4°C overnight. After 12- to 16-h incubation, membranes were washed with Tris-buffered saline containing Tween-20 (TBST) buffer and then incubated with secondary antibody. Specific bands were visualized using the enhanced chemiluminescence (ECL) detection kit (Advansta, CA, USA) and analyzed using FluorChem Q system (Protein Simple, CA, USA). The band intensities targeted to proteins were calculated and normalized to that of GAPDH using FluorChem Q system. Three experiments were repeated. The details of antibodies were shown as follows: anti-α-synuclein antibody (Santa Cruz Biotechnology, Santa Cruz, TX, USA), anti-Nurr1 antibody (Abcam, Cambridge, MA, USA), anti-GAPDH antibody (Cell Signaling Technology, Danvers, IL, USA), anti-NF-κB p65 antibody (Millipore), anti-Histone H1.4 antibody (Cell Signaling Technology), Goat anti-mouse IgG H&L (Proteintech Group Inc., Rosemont, IL, USA), Goat anti-rabbit IgG H&L (Proteintech Group Inc.), normal mouse IgG (Millipore), Goat anti-mouse Alexa 594 (Abcam), Goat anti-rabbit Alexa 488 (Cell Signaling Technology), and Hoechst 33342 (Cell Signaling Technology).

### RNA Extraction and Real-Time PCR

Total RNA was extracted using TRIzol reagent (Takara, Japan) and purified with chloroform. Two-microgram total RNA was used for cDNA synthesis (TransGene Biotech, Beijing, China) according to the manufacturer’s instructions. The mRNA levels of genes were analyzed by a SYBR Green RT-PCR kit (TransGene Biotech) with ABI Prism 7,500 Detection System (Applied Biosystems, Foster City, CA, USA). The experiment was repeated three times with three replicates per detection, and the relative expression levels of mRNA for genes were analyzed by the 2^−ΔΔCt^[ΔΔCt = (Ct_target gene_ − Ct_GAPDH_)_sample_ − (Ct_target gene_ − Ct_GAPDH_)_control_] formula (Livak and Schmittgen, 2001). The primers for real-time PCR assay are listed in Table 1 (Sangon Biological Engineering, Shanghai, China).

**Table 1:** Sequences of primers used in this article.

Names	Gene	Forward (5′ to 3′)	Reverse (5′ to 3′)
Real-time PCR	Nurr1	attccaggttccaggcaaac	agcaaagccagggatcttct
	TH	caatacaagcagggtgagcc	tagcatagaggcccttcagc
	AADC	gagctggagaccgtgatgat	ttagtccgagcagccagtagc
	DAT	tgtgaggcatctgtgtggat	tggaggtggtgatgattgca
	mouse-α-SYN	gctgctgagaaaaccaagca	accactgctcctccaacatt
	human-α-SYN	gagggtgttctctatgtaggct	tcaccactgctcctccaac
	GAPDH	tcgtggaaggactcatgacc	atgatgttctggagagcccc
Nurr1 promoter	−1,807/+199 bp	ctagctagctcgccgagcagcggcggcg	cccaagcttttaggagttctccgcgtctgt
	−853/+199 bp	ctagctagcgctgggtcacggtcactgt	cccaagcttttaggagttctccgcgtctgt
	−781/−594 bp	ctagctagcaacgtgggcactgcatgga	cccaagcttcatccccttttactccctttt
	−605/−418 bp	ctagctagcaaaaggggatgaaccgggt	cccaagcttgccggcgtggccaggtagtac
	−419/−233 bp	ctagctagccttgccttccgccggtgct	cccaagcttgcttgggtggtttttggtgt
	−233/−97 bp	ctagctagctggctaccaaggtgaacgca	cccaagcttggcccgccgcccaatcgccgc
ChIP	NF-κB	ttcgaagcgggaggaagag	acttgggcgttagatgggat

### Cell Treatment and Extraction of Cytoplasm and Nucleus Proteins

Cells were cultured with C-DIM12 (20 μM; Selleck, TX, USA) or actinomycin D (10 μg/ml; GlpBio Technology, CA, USA) for indicated times and then extracted with TRIzol reagent (Takara). Real-time PCR was used to detect the mRNA expression levels of targeted genes.

To detect the nuclear and cytoplasmic protein expression levels, the proteins were extracted with a nucleus and cytoplasm protein extraction kit according to the manufacturer’s instructions (Beyotime). The levels of cytosolic and nuclear proteins were analyzed by Western blot.

### Immunofluorescent Staining

Cells were seeded in a 24-well plate. After seeding for 24 h, cells were washed with PBS, fixed in 2% paraformaldehyde for 30 min, and permeated with 0.2% Triton X-100 for 30 min at room temperature. Then, cells were stained with the first antibodies overnight at 4°C. After washing with PBS, cells were incubated with fluorescent secondary antibodies for 1 h at room temperature. Nuclei were stained with Hoechst (1:10,000) for 5 min and washed with PBS three times. The fluorescence images were taken under an A1R MP multiphoton confocal microscope (Nikon, Japan).

### Flow Cytometry

For flow cytometry analysis, cells were washed with PBS and fixed with 2% paraformaldehyde for 30 min. Then, the first antibodies diluted in PBS with 1% saponin were used to stain the target proteins overnight at 4°C. After incubating with fluorescent secondary antibodies for 1 h at 4°C, cells were washed with PBS three times and analyzed by a FACSCanto II flow cytometer (Becton, Dickinson and Company, USA).

### Dual-Luciferase Reporter Gene Assay

The fragments of human Nurr1 promoter regions (−1,807/+199, −853/+199, −781/−594, −605/−418, −419/−233, and −233/−97 bp) were amplified by PCR using specific primers (Table 1), then cloned into pGL4.18 (Promega, WI, USA) luciferase reporter vector for generating the Nurr1 luciferase plasmids. The pGL4.18–Nurr1–promoter plasmids were confirmed *via* second-generation sequencing technology (Sangon Biological Engineering). Finally, α-SYN overexpressed cells were co-transfected with different pGL4.18–Nurr1–promoter luciferase vectors and pGL4.74 (hRluc/TK) vectors at 1 and 0.5 μg, respectively. After transfection for 48 h, the activities of firefly luminescence and renilla luminescence were detected with a Dual-Glo^®^ Luciferase Assay System (Beyotime). The firefly luciferase signal was normalized to the Renilla luciferase signal.

### Chromatin Immunoprecipitation

N2a stably over-expressed α-SYN cells were collected with a ChIP assay kit according to the manufacturer’s protocol (Beyotime). ChIP primer (Table 1) sets were checked for linear amplification and designed to amplify the regions of mouse Nurr1 promoter. Precipitated DNA was detected with real-time fluorescence quantitative PCR with ABI Prism 7500 Detection System (Applied Biosystems). The values were analyzed as percentage of input DNA using a calibration curve for quantification and normalized to control group (incubated with anti-IgG). Precipitated DNA was amplified with a DNA Polymerase Easy Taq^®^ kit (TransGene Biotech), and the amplified fragments were detected in 2% agarose gel.

### Statistical Analysis

All experimental results were expressed as the mean ± standard error of the mean (SEM). Statistical analyses were performed using SPSS 17 software (SPSS, Inc., USA). Each experiment was repeated three times. The significant differences were evaluated by one-way analysis of variance (ANOVA; GraphPad Inc., USA) and the difference was considered significant at a value *p* < 0.05.

## Results

### α-SYN Regulates the Expression of Nurr1

**Figure 1:**
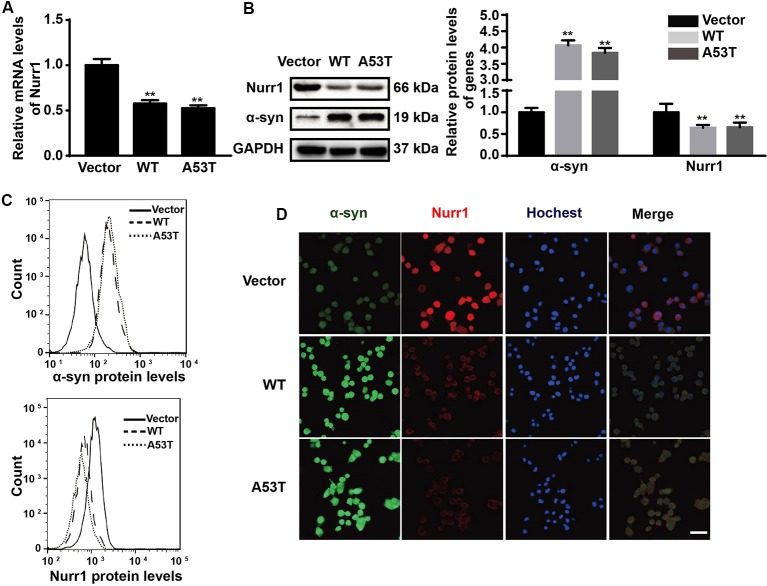
Impacts of α-synuclein (α-SYN)^WT^ and α-SYN^A53T^ on expression of nuclear receptor-related 1 protein (Nurr1). The mRNA and protein level of Nurr1 were detected in α-SYN (WT and A53T) stably overexpressed and control (Vector) N2a cells **(A,B)**. Flow cytometry was used to detect the levels of α-SYN and Nurr1 in the cultured cells as B **(C)**. The intracellular protein level of Nurr1 was further confirmed using immunofluorescent staining; α-SYN (green), Nurr1 (red), and nucleus were stained with Hoechst 33342 (blue). Scale bar: 50 μm **(D)**. Data were expressed as mean ± SEM. **p < 0.01 vs. control cells, *n* = 3.

In order to explore the effects of α-SYN on Nurr1 expression *in vitro*, we constructed N2a cells stably expressing α-SYN (WT and A53T) using pcDNA3.1(-)-SYNC or pcDNA3.1(-)-A53T plasmids. Real-time PCR was used to detect the mRNA level of Nurr1 in α-SYN overexpressed cells (WT and A53T). The results showed that Nurr1 expressions in α-SYN^WT^ and α-SYN^A53T^ overexpressed cells were significantly decreased to 57% and 53% compared with the vector control group, respectively (*p* < 0.01; Figure 1A). The protein levels of Nurr1 in both cell lines were also decreased by 35.7% and 34.3%, compared with the vector control group, respectively (*p* < 0.01; Figure 1B). Consistent with these data, the flow cytometry results further confirmed that the level of Nurr1 in α-SYN overexpressed cells was significantly reduced compared with that in control cells (Figure 1C). Moreover, the results of immunofluorescent staining demonstrated the same changed trend of Nurr1 expression as that of real-time PCR and Western blot analyses (Figure 1D). These results demonstrated that α-SYN can negatively regulate the expression level of Nurr1 *in vitro*.

### α-SYN Regulates the Nurr1-Targeted Downstream Gene Expressions

**Figure 2:**
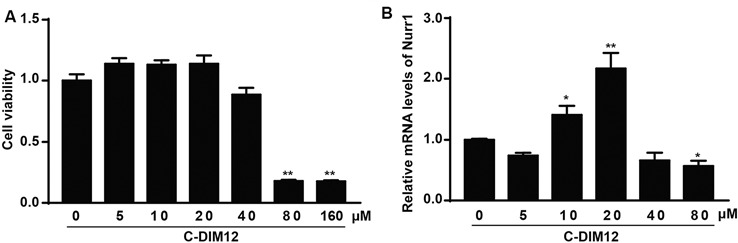
Cell viability and Nurr1 mRNA level in N2a cells cultured with C-DIM12. The cell viability and the mRNA level of Nurr1 were detected in N2a cells cultured with different concentrations of C-DIM12 **(A,B)**. Data were expressed as mean ± SEM. *p < 0.05, **p < 0.01 vs. control cells, *n* = 3.

**Figure 3:**
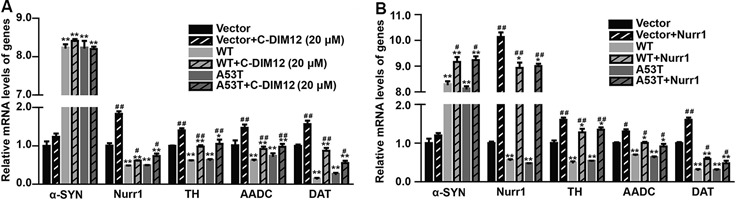
Effects of overexpression of α-SYN^WT^ and α-SYN^A53T^ on Nurr1 targeted genes *via* Nurr1. The mRNA levels of human and mouse α-SYN, Nurr1, Tyrosine hydroxylase (TH), AADC, and DAT were detected using real-time PCR in α-SYN (WT and A53T) stably overexpressed and control (Vector) N2a cells cultured with C-DIM12 (20 μM) for 6 h **(A)** or transfected with Nurr1 plasmids for 72 h **(B)**. Data were expressed as mean ± SEM. *p < 0.05, **p < 0.01 vs. control cells, ^#^*p* < 0.05, ^##^*p* < 0.01 vs. non-C-DIM12 cultured or non-Nurr1 transfected cells, *n* = 3.

To further assess the impacts of α-SYN on Nurr1-mediated DA function, we cultured α-SYN overexpressed cells with a synthetic Nurr1 activator C-DIM12 (Hammond et al., 2015), which can induce significant elevation in Nurr1 mRNA level at non-toxic concentrations (Figures 2A,B). Real-time PCR was used to detect the expression levels of Nurr1-targeted downstream genes including tyrosine hydroxylase (TH), dopa decarboxylase (AADC), and DA transporter (DAT) in these cells. We found that overexpression of α-SYN down-regulated these gene expressions (*p* < 0.01; Figure 3A). As expected, after 6 h of C-DIM12 exposure, restorations of Nurr1-targeted gene expression were observed in C-DIM12-treated cells (*p* < 0.05; Figure 3A). Additionally, we overexpressed Nurr1 in α-SYN overexpressed cells. The results showed that all changes of Nurr1-targeted gene expressions in α-SYN overexpressed cells can be reversed by transfection of Nurr1 overexpression plasmids (*p* < 0.01; Figure 3B). All these results demonstrated that α-SYN regulated the Nurr1-targeted downstream gene expressions *via* Nurr1 and further verified the modulation of α-SYN on Nurr1.

### α-SYN Affects the Transcriptional Activity of Nurr1 Promoter

**Figure 4:**
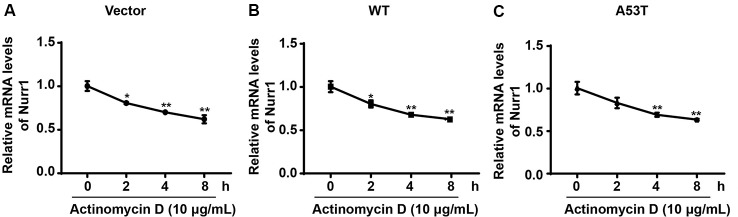
α-SYN did not regulate the mRNA stability of Nurr1. Control cells (vector), α-SYN^WT^ (WT) and α-SYN^A53T^ (A53T) stably overexpressed cells were cultured with actinomycin (10 μg/ml) for 0, 2, 4, and 8 h, and then detected the mRNA levels of Nurr1 in these cells (**A–C**). Data were expressed as mean ± SEM. *p < 0.05, **p < 0.01 vs. control cells, *n* = 3.

To explore the mechanism of α-SYN on Nurr1 transcription level, we first detected the regulation of α-SYN on Nurr1 mRNA stability. α-SYN overexpressed (WT and A53T) cells were cultured with an RNA polymerase inhibitor (actinomycin D, 10 μg/ml) for 0, 2, 4, and 8 h. Real-time PCR analyses showed that α-SYN did not significantly alter the mRNA stability of Nurr1 in either α-SYN^WT^ or α-SYN^A53T^ transfected cells compared with control cells (Figures 4A–C).

**Figure 5:**
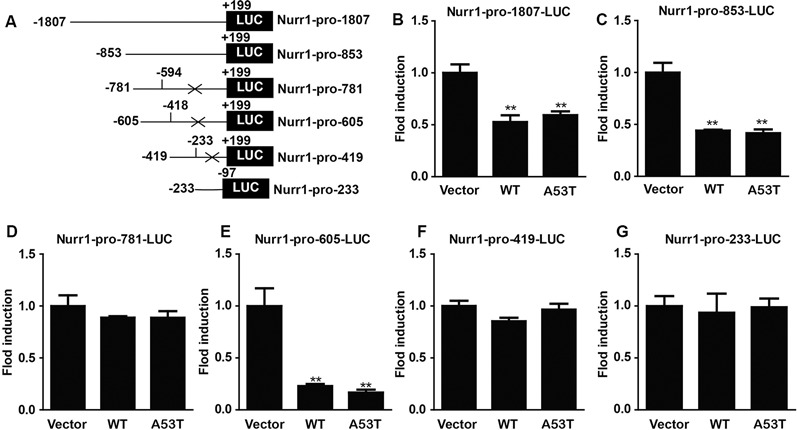
α-SYN regulated the transcription activity of Nurr1. **(A)** The schematic diagram of different Nurr1 promoter fragment luciferase plasmids. Dual-Glo luciferase assay determined the transcription activities of Nurr1 different promoter regions (−1,807/+199, −853/+199, −781/−594, −605/−418, −419/−233, and −233/−97 bp) in control cells (vector), α-SYN^WT^ (WT) and α-SYN^A53T^ (A53T) transfected cells **(B–G)**. Data were expressed as mean ± SEM. **p < 0.01 vs. control cells, *n* = 3.

Then, we constructed luciferase plasmids contained with different fragments of Nurr1 promoter (−1,807/+199, −853/+199, −781/−594, −605/−418, −419/−233, and −233/−97 bp; Figure 5A) and co-transfected them with pGL4.74 (hRluc/TK) plasmids into α-SYN overexpressed cells, respectively. The relative activities of these luciferase plasmids were detected and analyzed (Figures 5B–G), showing that overexpression of α-SYN significantly down-regulated the transcription activities of Nurr1–promoter luciferase plasmids contained with Nurr1–pro-1807, Nurr1–pro-853, and Nurr1–pro-605 (*p* < 0.01; Figures 5B,C,E). Compared to these three Nurr1–promoter luciferase plasmids, we identified the common Nurr1 promoter region ranging from −605 bp to 418 bp, indicating that overexpression of α-SYN reduced the transcription activity of Nurr1 promoter.

### α-SYN Regulates the Transcription Activity of Nurr1 *via* NF-κB

**Figure 6:**
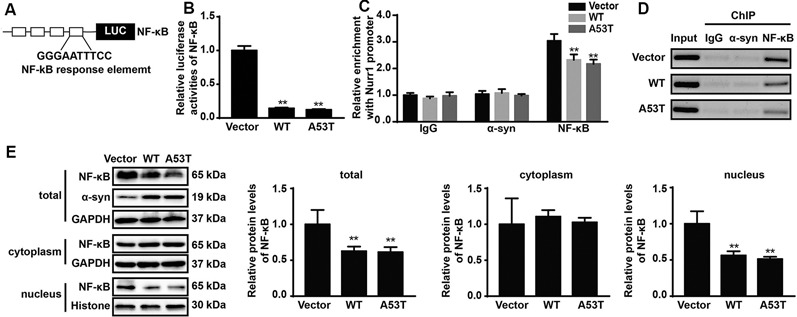
α-SYN regulated the transcription activity of Nurr1 *via* NF-κB. **(A)** The schematic diagram of NF-κB binding site luciferase plasmid. Dual-Glo luciferase assay determined the transcription factor activity of NF-κB in control cells (vector), α-SYN^WT^ (WT) and α-SYN^A53T^ (A53T) transfected groups **(B)**. ChIP-real-time PCR assay examined NF-κB or α-SYN occupancy of Nurr1 promoter in α-SYN overexpressed cells **(C)**. The amplified fragments were detected by 2% agarose gel **(D)**. After cytoplasm nucleus extraction, the nucleus and cytoplasm **(E)** protein expression of NF-κB was detected by Western blot. Data were expressed as mean ± SEM. **p < 0.01 vs. control cells, *n* = 3.

Using JASPAR database (Fornes et al., 2020), we analyzed the Nurr1 promoter region ranging from −605 bp to −418 bp and found that this region contained the binding site of NF-κB, which is identified as the highest score in bioinformatics analysis results. This led us to speculate that α-SYN regulated the expression of Nurr1 possibly *via* NF-κB. To confirm our speculation, we first constructed a luciferase plasmid containing four repeated binding sites of NF-κB (Figure 6A) and co-transfected it with pGL4.74 (hRluc/TK) plasmids into α-SYN overexpressed cells. The results of dual-luciferase reporter gene assay showed that overexpression of α-SYN significantly inhibited the transcription factor activity of NF-κB (*p* < 0.01; Figure 6B).

We further performed ChIP assay to detect the interaction between α-SYN and α-SYN-regulated Nurr1 promoter region. Our data indicated that α-SYN showed no direct interaction with this region whether in control cells or in α-SYN overexpressed cells. However, ChIP assay illustrated that α-SYN reduced the NF-κB binding quality with Nurr1 promoter (Figures 6C,D).

**Figure 7:**
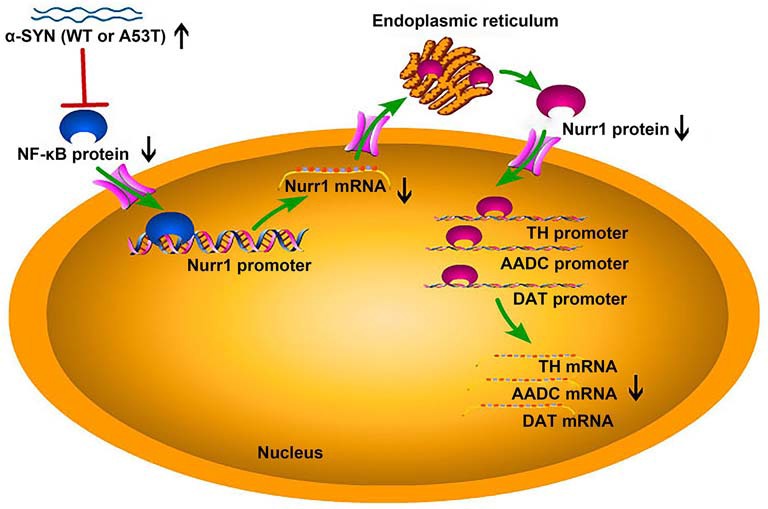
The schematic diagram of this study. Overexpression of α-SYN^WT^ (WT) and α-SYN^A53T^ (A53T) reduced NF-κB protein level and inhibited the binding quantity of NF-κB with Nurr1 promoter, thereby decreasing Nurr1 and its target genes (TH, AADC, and DAT) mRNA level.

To further confirm the involvement of NF-κB in α-SYN-regulated Nurr1 expression, we determined the expression level of NF-κB in α-SYN overexpressed cells. Western blot analysis suggested that both α-SYN^WT^ and α-SYN^A53T^ induced dramatic down-regulation of NF-κB (*p* < 0.01; Figure 6E). In addition, we separated nucleus–cytoplasm proteins and analyzed the expression levels of NF-κB. The Western blot data indicated that α-SYN down-regulated the nucleus protein level of NF-κB as much as the total protein (*p* < 0.01), whereas the cytoplasm protein level of NF-κB was not significantly changed by overexpression of α-SYN (Figure 6E). Taken together, our results demonstrate that the α-SYN-induced down-regulation of Nurr1 expression might be through the nucleus NF-κB-related pathway, thereby affecting the expression of DA-associated genes (Figure 7).

## Discussion

α-SYN is expressed in many cells and tissues (Dev et al., 2003; Lin et al., 2012). Mutation and overexpression of α-SYN can cause PD, but it is unclear why mutation and overexpression of α-SYN mostly affect DA neurons in the substantia nigra (Decressac et al., 2012). Previous studies have shown that Nurr1 is a critical modulator for the development and function of midbrain DA neurons (Jankovic et al., 2005; Kadkhodaei et al., 2009, 2013; Montarolo et al., 2019), and regulates genes of the DA signaling pathway, including TH, AADC, and DAT (Saucedo-Cardenas et al., 1998; Kadkhodaei et al., 2009; Raina et al., 2020). In this study, we have demonstrated that α-SYN can reduce several DA-associated gene expressions *via* its down-regulation on Nurr1 (Figures 3A,B), indicating that the suppression of Nurr1 expression by α-SYN overexpression is an important molecular basis for DA neuron dysfunction. We have then explored of how α-SYN affects Nurr1 expression and its function, the results of which may help understand the selective impacts of α-SYN on DA neurons and provide new strategies to counteract against α-SYN-induced DA neurons degeneration.

α-SYN is partly located in the nucleus, and the binding of α-SYN with nuclear chromatin has been found in the nigra of PD patients’ brain, supporting its pathological association (Siddiqui et al., 2012). From our immunofluorescent study, it is obvious that α-SYN is located in both nucleus and cytoplasm, and Nurr1 is mostly localized in the nucleus (Figure 1D). It is speculated that Nurr1 is one of the α-SYN targets and may contribute to the pathological process of PD (Decressac et al., 2013). Reduced expression of Nurr1 has been detected in either WT or A53T α-SYN over-expressed neuroblastoma cell lines (Baptista et al., 2003). Moreover, Lin et al. (2012) have explored the mechanism of α-SYN on Nurr1 expression and found that overexpression of α-SYN (WT and A53T) promotes the proteasome-associated degradation of Nurr1 protein. It is suspected that there may exist a feedback loop between α-SYN and Nurr1 (Devine, 2012). The results from our study (Figure 1A) are consistent with other reports conducted in cell lines and animal model (Baptista et al., 2003; Decressac et al., 2012; Lin et al., 2012). In our study, we have first demonstrated that overexpression of α-SYN (both WT and A53T) does not disturb Nurr1 mRNA stability (Figure 4), but influences the transcription activity of Nurr1 promoter ranging from −605 bp to 418 bp (Figure 5B), indicating that α-SYN can affect the mRNA synthesis of Nurr1. All of these findings may help understand the role of Nurr1 in α-SYN-mediated PD pathological process and may lay the foundation to study the feedback loop between α-SYN and Nurr1.

Previous studies have shown that overexpression of α-SYN inhibits NF-κB expression, up-regulates GSK3β protein in neurons, suggesting that the pathological effects may be mediated by NF-κB signaling pathway (Yuan et al., 2008). Recent studies have also demonstrated that α-SYN significantly inhibits the activity of NF-κB (Reynolds et al., 2008). Furthermore, α-SYN interacts directly with microglia to induce NF-κB accumulation and downstream chemotactic factor expressions (Cao et al., 2012). The stimulation of E-type prostaglandin receptor 1 (EP1) by prostaglandin E2 (PGE2) up-regulates the expression of Nurr1 *via* a mechanism involving the activation of NF-κB signaling pathways (Ji et al., 2012). NF-κB has been suggested to be a transcriptional factor of Nurr1 and mediates Nurr1 mRNA synthesis (McEvoy et al., 2002). Pro-inflammatory mediators contained with IL-1β, TNF-α, and PGE2 can also enhance the binding activity of NF-κB with Nurr1 promoter, thereby promoting Nurr1 transcription and elevating Nurr1 mRNA and protein level in primary rheumatoid arthritis and normal synoviocytes (McEvoy et al., 2002). Here, we demonstrate that NF-κB is the highest score transcription factor in the bioinformatics analysis of α-SYN-regulated Nurr1 promoter region, and overexpression of α-SYN inhibits NF-κB binding quality with Nurr1 promoter (Figures 6C,D). Moreover, the protein level of NF-κB, especially the nuclear NF-κB, is down-regulated by α-SYN overexpression (Figure 6E), which is consistent with the previous findings that overexpression of α-SYN can regulate the NF-κB-associated pathway (Cao et al., 2012). However, we cannot rule out other potential transcription factors or intermediate molecules involved in the mechanism of α-SYN on Nurr1 synthesis.

In summary, α-SYN is expressed in many tissues and cells, and mutation or overexpression of the gene selectively affects the DA neuron function probably through the effects on NF-κB-mediated Nurr1 synthesis. Our work may help uncover the molecular mechanism of α-SYN on DA neuron degeneration in association with PD and may help find the potential molecular targets for PD therapy.

## Data Availability Statement

All datasets generated for this study are included in the article.

## Author Contributions

CJ, HQ, and CC performed the experiments. CJ, HQ, XW, and ZY collected and analyzed the research data. CJ, HQ, and WL drafted the manuscript. WL and SC revised the manuscript. WL and HC conceived and designed the experiments. All authors read and approved the final manuscript.

## Conflict of Interest

The authors declare that the research was conducted in the absence of any commercial or financial relationships that could be construed as a potential conflict of interest.
